# Bioinspired super-hydrophobic fractal array *via* a facile electrochemical route: preparation and corrosion inhibition for Cu†

**DOI:** 10.1039/d1ra06473h

**Published:** 2021-12-20

**Authors:** Robert H. B. Miller, Yinsha Wei, Cong Ma, Longyun Li, Jihan Shao, Shugang Hu, Sonkarlay J. Y. Weamie

**Affiliations:** College of Safety and Environmental Engineering, Shandong University of Science and Technology Qingdao 266590 P. R. China roberlynehbmiller@gmail.com husg8921@163.com +8619153242137; College of Engineering, University of Liberia 1000 Monrovia 10 Liberia; School of Computer Science and Engineering, Hunan University Changsha Hunan China

## Abstract

Super-hydrophobic surfaces (SHS) usually are formed from a combination of low surface energy materials and micro/nanostructures *via* two-step approaches, and they have promising applications in material corrosion protection. In this paper, the authors obtained a super-hydrophobic surface onto the copper plates through a rapid one-step electrodeposition process from the electrolytic solution containing cobalt nitrate (Co(NO_3_)_2_·6H_2_O), myristic acid, and ethanol. The electrochemical impedance spectroscopy and polarization curve are adopted to evaluate a super-hydrophobic surface's durability and corrosion resistance. The results demonstrate that the super-hydrophobic cobalt myristate coating showed excellent corrosion inhibition in simulated seawater solution with a corrosion inhibition efficiency as high as 98.82%. Furthermore, the super-hydrophobic layer could be considered a barrier and thus require an ideal air-liquid interface that inhibits the diffusion of the corrosive species. The construction of super-hydrophobic characters with a self-cleaning property is significant and used widely, attracting numerous studies for obtaining surfaces with low surface energy and micro/nanostructures. The as-fabricated super-hydrophobic surfaces possess the external surface adhesive force to the water phase and excellent self-cleaning and antifouling ability. By adjusting processing time, the water contact angle of the coated copper surface reaches 152.9°, showing a superb superhydrophobicity. The morphology, chemical composition, and wettability characterization were analyzed using scanning electron microscopy (SEM), X-ray photoelectron spectroscopy (XPS), and contact angle measurements. In addition, a scanning Kelvin probe (SKP) usage in this work is to measure the atmospheric corrosion behavior of copper with the super-hydrophobic coating. Thus, this proposed method provides a simple way to rapidly equip super-hydrophobic coating onto the metal surface to realize corrosion inhibition.

## Abstract 2

Super-hydrophobic surfaces (SHS) usually are formed from a combination of low surface energy materials and micro/nanostructures *via* two-step approaches, and they have promising applications in material corrosion protection.

## Graphical abstract

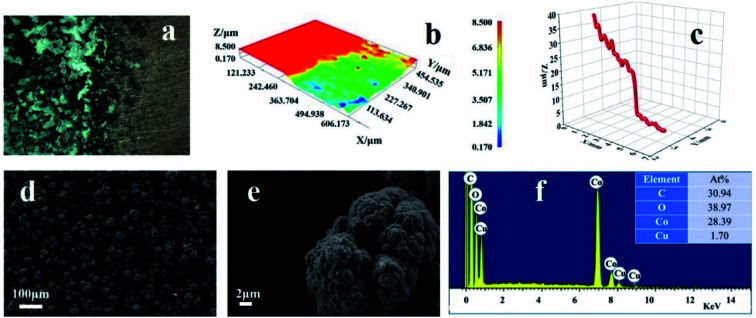

## 1 Introduction

Copper is an engineering material, and it has broad field applications, including heat conductors, constructional materials, electrical power lines, water supply pipelines, and the defense industry.^1^ Based on the application of non-ferrous metal, copper is next to aluminum. Most of the conductive materials used in the electrical and electronic industries dominate copper, which usage is in cables and circuit boards.^2^ In industrial plants and facilities, *e.g.*, power plants and ships, copper and alloys are widely employed to produce valves, heat exchangers, and pump systems.^3^ As a result, copper is highly essential in human society and daily life. However, it is not inert enough to the external environment. It may corrode directly or indirectly when it meets corrosive species during the practical service,^4^ especially in a wet climate.

Therefore, researchers hypothesized that corrosion resistance might increase significantly by reducing the contact area between the copper substrate and corrosive liquids.^5^ The unavoidable corrosion phenomenon will cause consequences, such as the electrical board's failure and the heater's emission, making corrosion the chief criminal to collapse instruments and facilities.

Globally, bioinspired super-hydrophobic surfaces have currently generated considerable interest in academic research and future industrial applications due to their numerous benefits, including self-cleaning capabilities,^6^ drag reduction,^7^ water/oil separation,^8^ and corrosion resistance.^9^ Super-hydrophobic surfaces typically have a water contact angle (WCA) greater than 150° and exhibit either the lotus effect (rolling angle (RA) less than 10°) or the pinning effect (no RA or RA more than 10°).^10,11^ According to studies on wild organisms with super-hydrophobic surfaces, the presence of micro/nanostructured surface morphology is the primary factor responsible for developing this unique surface wettability.^12,13^ Additionally, some unique chemical components contribute to forming a bioinspired super-hydrophobic surface.^14,15^ Therefore, two elements inspired by nature are to create superhydrophobicity: (i) a rough surface texture with a unique binary structure and (ii) a change in surface chemistry *via* a low-free-energy coating.^16^

Super-hydrophobic surfaces may also protect materials in the seawater phase. This coating utilizes the “air cushion” included within the super-hydrophobic matrix to block the passage of seawater to the metal phase beneath. Corrosion is impossible to occur on metal surfaces if seawater and dissolved oxygen do not contact them. There are many approaches to creating rough micro/nanostructures on metal substrates when added to super-hydrophobic layering.^17–21^ These techniques include electrodeposition and laser surface texturing, among others. For the time being, several methods have been used to coat the surface of copper with a super-hydrophobic coating. It was divided into two types of processes: one-step techniques and two-step procedures, described below. The conventional anodic dissolution–deposition approach^22^ employed is for the one-step method.^23–26^ With this approach, it is possible to attach complexes that contain a hydrophobic ligand and a metal ion to the surface of a metal substrate. Typically, the hydrophobic ligand is a long chain organic molecule. The solid-state deposit will build onto the metal substrate *via* the continuous electrolysis process. The acquiring of super-hydrophobic coating wettability is due to the deposit's roughness. However, this procedure has an inherent drawback, in which building-up is required for the new metal. Where the sacrificing of more metals creates the coating. The usage of coatings protects metals against corrosion. If the layer requires metal consumption during construction, it cannot be considered a good option for material protection. As a result, it lacks practical utility. The two-step technique is motivated by the necessity for superhydrophobicity, which requires both surface roughness and hydrophobicity for the air cushion to store inside the matrix. Typically, the initial stage in this method is to create the rough surface, followed by the modification of the surface with the hydrophobic moiety. The perceived contact angle will perform at a high value following the adjustment. It is, however, tiresome and time-consuming. As a result, discovering simple, acceptable one-step techniques that do not require the use of much metal is still highly desired from both academic and industrial perspectives.

This study generates a bioinspired super-hydrophobic cobalt myristic acid complex coating with micro/nanostructure on a Cu substrate utilizing a single one-step electrodeposition process. The electrodeposition procedure used to create the coating is not costly, and the layer is entirely composed of cobalt myristic acid combinations. The rough topology enables super-hydrophobic wettability. The metal is not consumed during the coating construction process, as is the case with those mentioned above traditional dissolution–coordination–deposition mechanisms described in previous works. The building of the super-hydrophobic coating is by using a coordination–deposition agent rather than the metal to be protected. The super-hydrophobic film's form, chemical content, and wettability, and the coating's self-cleaning ability were studied using SEM, energy-dispersive spectroscopy (EDS), XPS, and water contact angle (WCA) measurement. The stability of the coating was analyzed using sand abrasion testing. The results reveal that the deposited coating consisted of cobalt crystals and myristate, significantly affecting the surface free energy. Therefore, it was necessary to investigate the electrodeposited super-hydrophobic layer's surface and chemical composition to determine the formation mechanism. When utilized as a target coating, the super-hydrophobic surface also possesses anti-adhesion properties.

Meanwhile, the super-hydrophobic coating has a high level of corrosion prevention due to the existence of an air cushion on the Cu surface. As a result, the one-step bioinspired technique's super-hydrophobic Co-myristic acid complex has the potential to be employed in saltwater for corrosion and bionic suppression. Therefore, the electrodeposition procedure is straightforward, inexpensive, and successful, making it viable for mass-producing bioinspired super-hydrophobic surfaces on various metals.

## 2 Experimental

### 2.1 Sample preparation

Copper (Cu, purity 99%, geometry dimension 30 mm × 15 mm × 0.3 mm) polishing was by 800, 1500, and 2000 mesh sandpaper and ultrasonically washed in ethanol and deionized water in sequence. The electrochemical deposition was realized in two-electrode configuration electrolysis using a direct current source (MS-603D) at room temperature. The electrochemical deposition took two Cu plates of the same area as cathode and anode. The mixed solution containing 0.05 M Co(NO_3_)_2_·6H_2_O and 0.1 M myristic acid dissolved in ethanol (Shanghai Maclin Biochemical Technology Co., Ltd.) usage was an electrolyte with a volume of 50 ml. During the preparation of the solution, the procedure was as follows: the myristic acid was firstly dissolved in ethanol to get a mixture. After that, the inorganic salt was further dissolved into the as-prepared solution to form a homogeneous solution. The electrodeposition took 10 V for the 1200 s with approximately 2 cm between the two electrodes. When the electrodeposition process ended, we removed the sample immediately from the solution. Then, dipped in ethanol for slight abstersion. During this process, the washing of the material repetitively may eventually lead to coating failure. Then, the deposit was dried naturally. High-temperature drying was avoidable during the drying of the residues. Finally, we stored the as-prepared samples in a vacuum chamber for further experiments.

### 2.2 Morphology and composition analysis

Knowing the surface morphology is the first step to understanding the deposit. In this report, the revealing of the morphology of samples after electrodeposition from the micrometer and nanometer scale microscope shows the excellent appearance and for roughly knowing the surface morphology, two and three-dimensional topology of the conjunction area with bare Cu and Cu covered by deposit characterizing by optical microscopy (Hirox 8700). Field emission electron microscopy (FE-SEM, Zeiss, ultra-55, beam energy of 5.0 kV) further discloses and understands the deposit's delicate structure. The energy-dispersive spectroscopy (EDS, Oxford Instruments, X-max) technique roughly indicates the composition. Additionally, the characterizing of the chemical bonding of the sample obtained on the cathodic deposition was by X-ray photoelectron spectroscopy (XPS, Thermo Fisher Scientific, 250xi).

### 2.3 Wettability, self-cleaning, and mechanical robustness property evaluation

The determination of the contact angle was by a contact angle measuring instrument. At the typical probe, the volume of the water drop was about four μL. During the test, the selected area for conducting the contact angle measurement was made horizontal. After dripping the droplet onto the surface, the digital camera recorded the wetting state, and the embedded software calculated the contact angle. We evaluated the average value at different positions. The usage of the high-speed camera was to record the entire dynamic process of the droplet to the substrate surface, including the movement of falling, impacting, and leaving the sample surface. The frame rate and shutter speeds were 10 000 fps and 1/256 000 s, respectively. The free fall height of the water droplet to the target surface was 4 cm. The process of the self-cleaning experiment was as follows: firstly, sprinkle zinc oxide powder distribution was on the inclined surface (inclined as 5.4°). Then, a syringe was adopted to a drip water droplet on the as-prepared deposit slowly. During the washing process, using a digital camera recorded the removal process of dust by water droplets.

On the other hand, the mechanical robustness property is an essential aspect for practical usage. With sandpaper (1000 mesh) as the abrasive medium, we simulated the actual friction environment. During the evaluation process, the deposit surface was directly in direct contact with the sandpaper, and a weight of 50 g was placed on the sandpaper as a load to increase the mechanical destruction effect. The copper-covered deposit moved by a step of 5 cm on the sandpaper, and the measurement of the contact angle of the film was by a contact angle measuring instrument. The repeatability of each experiment for measuring contact angle was at least three times to ensure the accuracy of the data. To further evaluate the robustness and stability of the coating, two samples were placed in a 1000 ml of deionized water contained in a beaker for 2 days at ambient temperature. The samples were analyzed after several 24 hours, and contact angles were measured using a contact angle measurement aided by the Image-Pro software, and the impact in crystal morphology was analyzed using an optical microscope (Hirox 8700). Each sample was measured four times to get excellent precision.

### 2.4 Investigating the coating diatom adhesion in seawater

Biological fouling is yet another type of degrading effect caused by the natural marine environment that might occur. Because the as-emitted Cu ion from corrosion can act as an effective chemical antibacterial when Cu metal is in its bare state, the biofilm from the natural seawater environment is suppressed when Cu metal is in its bare condition. Therefore, finding the most effective approach for shielding Cu metal from biofouling rather than devouring the metal is of critical importance. In this investigation, the ability of the theorized super-hydrophobic with microbes was tested successfully, and the results were promising. The organism *Sellaphora atomoides* was chosen as the group's representative because of its unique characteristics. Following the technique established in the previous study,^27^ collecting and culturing the diatom *Sellaphora atomoides* were carried out. Simply said, the cultivated algae suspension was transferred to a pre-sterilized glass vessel to evaluate whether bacteria were present on the bare Cu and super-hydrophobic Cu surfaces after being immersed in the solution. From a statistical standpoint, a total of two parallel samples were produced and placed in diatom suspension for cells to attach to achieve the average biofilm status from two similar samples. After being submerged for ten days, the soaking materials, which included bare Cu and super-hydrophobic Cu, were removed from the vessel and examined for the presence of a biofilm. Preparation of the sample surface for examination with an epifluorescence microscope (Leica, Germany) included gentle washing with natural seawater and diluted in an ethanol solution before analysis with the microscope. The extent to which the organisms covered the surface of the samples was determined using six visual fields on each parallel sample. It was possible to quantify the biofilm status with the help of the Image-Pro software.

### 2.5 Corrosion resistance property

Scanning Kelvin probe (SKP) technology, as one of the most advanced and sophisticated electrochemical methods, can detect the potential voltaic distribution of the metal surface. SKP is intrinsically a non-contact detecting method, which found small changes in the system interface *in situ*. This principle predicts the spatial distribution of the redox reaction and the quantification information of metal corrosion. Focusing on the local and micro-corrosion perspective, SKP (VersaSCAN, Ametek) directly detects the change of sample surface potential after electrodeposition. In this study, the scanning rate adopted was 200 μm s^−1^.

A three-electrode working fashion measured the electrochemical impedance (EIS) and Tafel curves. The usage of the samples of bare Cu metal and Cu covered by the deposit was the working electrode, with the exposure area of the working electrode about 1 cm^2^. The saturated calomel electrode (SCE) and platinum plates were the reference and counter electrodes in the cell, respectively. The open-circuit voltage of the electrode was the starting voltage for EIS measuring. And the frequency range was set to 0.01 to 105 Hz with an interference potential of 10 mV.

Meanwhile, the selection of the sensitivity was 5 mV. The test medium was an aqueous solution containing 3.5 wt% NaCl. We used the ZsimpWin software to fit the electrochemical parameters of the as-obtained data and figure out equivalent electrical circuits. The voltage range was between −0.25 V and 0.25 V for obtaining the Tafel curve, and the scanning speed was 1 mV s^−1^.

## 3 Results and discussion

**Fig. 1:**
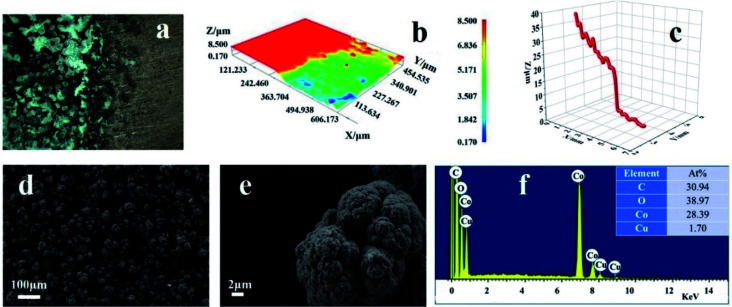
(a) The optical image illustrates the comparison between the deposit and the original copper substrate. (b) A three-dimensional height diagram of the coating. (c) The height plot corresponds to a cross-section indicated in (b). (d and e) SEM images of the as-fabricated deposit on copper. (f) The EDS of the deposit. The inset table corresponds to the content of the elements. All achievement of deposition was at 10 V with an electrolysis time of 20 min.

As illustrated in Fig. 1, the preparation of the super-hydrophobic surface is by one-step electrodeposition in a mixed solution of ethanol containing cobalt nitrate and myristic acid. The different microscopy techniques reveal the surface morphology of the prepared cathode surface. As shown in Fig. 1a–c, the two-dimensional and three-dimensional topology of the coating and the thickness are revealed by using optical microscopy. The coating on the cathode is brightly black and based on the macroscopic perspective, and the as-formed deposit is very homogeneous on the substrate. Fig. 1b and c demonstrate that the thickness of the layer is *ca.* 25 μm. The optical microscopy intuitively gives the appearance of the deposit. SEM image will afford the fine structure of the residue obtained *via* the electrolysis. From Fig. 1d, the micro–nano form on the cathode surface presents a uniform fractal spherical shape.

The high-magnification SEM image is illustrated in Fig. 1e, revealing that the spherical shape is multi-scale and uneven. In addition, there is a gap existing among the fractals, using to store the air. Therefore, it suggests that the formation of the spherical structure is from a discrete electrolytic solution containing 0.05 M cobalt nitrate and 0.1 M myristic acid, and the electrodeposition is conducted at a DC voltage of 10 V for 20 min. As shown in Fig. 1f, the prepared coating mainly consists of C, O, Co, and Cu elements through EDS analysis. The Cu element comes from the Cu substrate, and the origination of Co^2+^ is from the deposit *via* the electrolysis of cobalt nitrate precursor solution, while C and O are mainly from myristic acid.

**Fig. 2:**
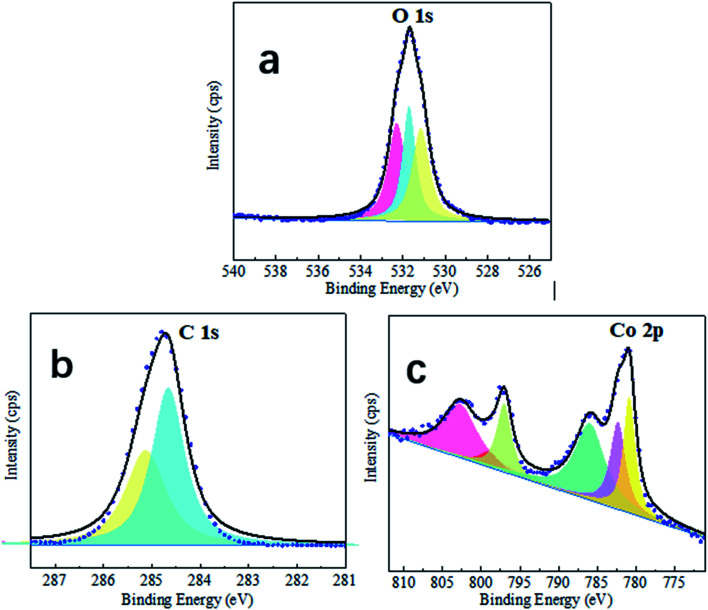
The performed XPS high-resolution single peak analysis for the (a–c) O 1s, C 1s, and Co 2p, respectively.

The surface chemistry of the sample was analyzed using XPS and EDS. As illustrated in Fig. 2, XPS spectra can provide additional information about surface chemistry. The XPS survey spectrum of the fabricated super-hydrophobic coating revealed three peak signals of C 1s, O 1s, and Co 2p. C/O/Co has an atomic ratio of 28 : 4 : 1, which is consistent with the cobalt myristate stoichiometry (Co[CH_3_(CH_2_)_12_COO]_2_). Notably, the as-prepared surface contained the highest concentration of carbon per percentage according to the stoichiometry provided, followed by oxygen and cobalt. Therefore, we hypothesize that the formed cobalt myristate increased surface carbon content and that the superhydrophobicity is due to the nonpolar functional groups of C–C(H) introduced by the cobalt myristate. This conclusion is consistent with previous research: increasing the carbon content increases the hydrophobicity of the surface due to the presence of low-free-energy functional groups such as CH_2_ and CH_3_.^28,29^

**Fig. 3:**
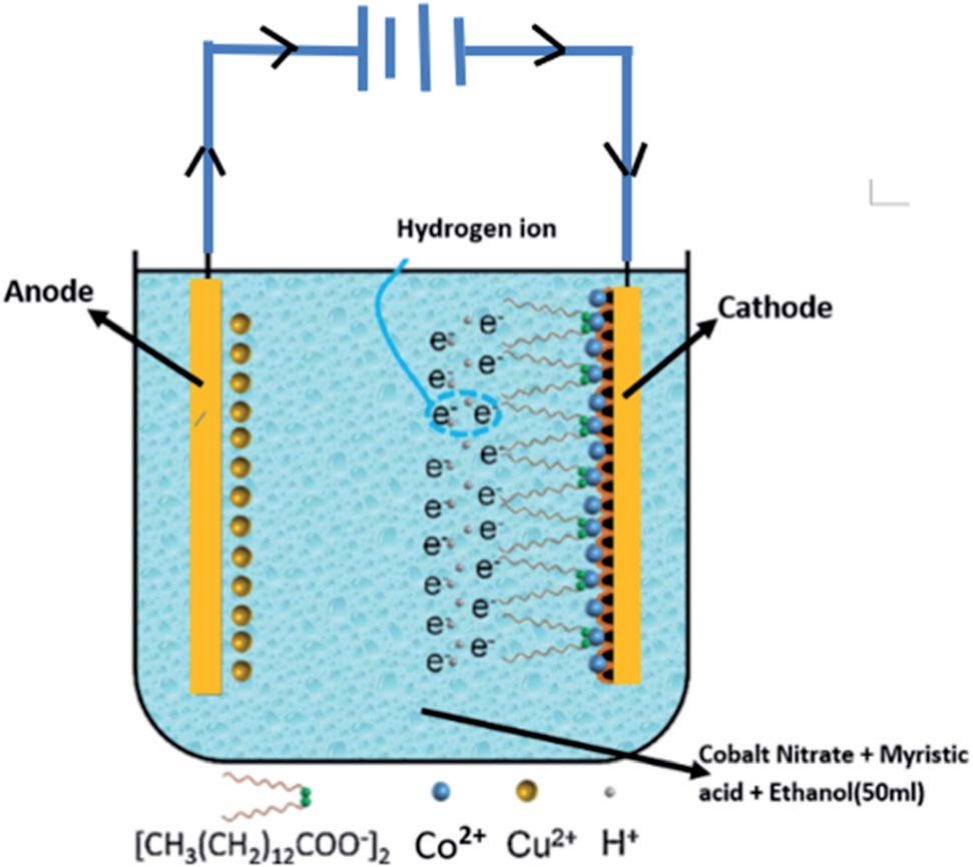
Schematic illustration of the electrodeposition process for the fabrication of super-hydrophobic cobalt film.

Additionally, residual distilled water exists. Following the electrodeposition process, the surfaces were cleaned with distilled water and dried in the ambient air. Thus, the hydroxylation of the newly formed cobalt ion would be because of the moist environment.^30^ The illustration of the high resolution of the C 1s spectrum is in Fig. 2b, and two firm peaks at 284.7 and 287.0 eV were identified as C–C(H) and O=C–O moieties, respectively, confirming that the long-chain molecules of myristic acid were attached to the as-prepared cobalt film.^31^ First, the appearance of the Co^2+^ peak confirms the existence of cobalt ions in the coating. Second, Fig. 2c demonstrates the Co 2p spectrum's excellent resolution. The highest peak in the range at 781 eV is due to Co 2p.^32^ The surface chemistry analyses reveal that the as-prepared super-hydrophobic coating with protrusive clusters consists of cobalt myristate (Co[CH_3_(CH_2_)_12_COO]_2_) species. Fig. 3. Shown the possible formation mechanism. Co ions (Co^2+^) would move to the cathodic electrode and quickly get an electron to generate cobalt myristic under the applied DC voltage between the two copper electrodes. The freshly formed cobalt ion contributed to the anisotropy crystal growth of cobalt myristate. Therefore, the appearance of cobalt myristate (Co[CH_3_(CH_2_)_12_COO]_2_) resulted from the reaction of cobalt ion and myristic acid under the application of DC voltage, which led to the presence of low-surface-energy functional groups (–CH_3_ and –CH_2_) on the as-prepared super-hydrophobic surface.

Meanwhile, some hydrogen ions (H^+^) around the cathodic plate also gained electrons to produce the hydrogen (H_2_) during the electroplating process. The released gas led to the loose morphology on the obtained super-hydrophobic surface. Once the liquid droplet placement is on the surface, many air pockets would be trapped underneath the droplet due to this unique surface structure, resulting in super-hydrophobic performance. The following formulas explain the whole reaction process on the cathodic electrode:

1 Co^2+^ + 2e^−^ → Co

2 Co^2+^ + 2CH_3_(CH_2_)_12_COOH → Co[CH_3_(CH_2_)_12_COO]_2_ + 2H^+^

3 2H^+^ + 2e^−^ → H_2_(g)

**Fig. 4:**
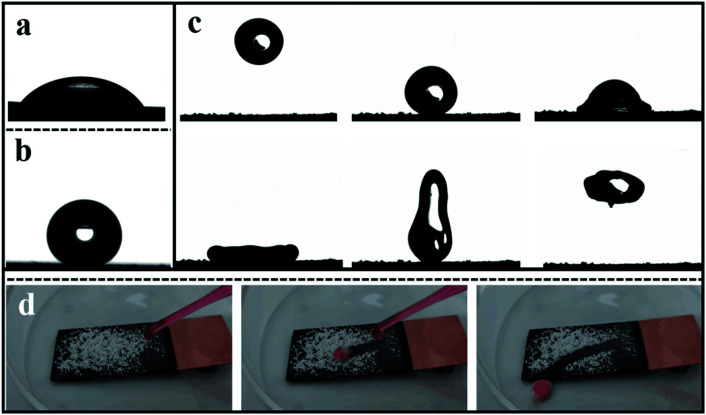
Wettability and self-cleaning capability evaluation. (a and b) Wettability measurement of bare Cu and deposit on Cu. (c) Dynamic motion of water droplet to the super-hydrophobic surface using a high-speed camera as the monitoring tool. (d) Self-cleaning effect of the superhydrophobic surface.

The use of the contact angle measurement is to describe the wettability of the material. As displayed in Fig. 4a, the contact angle of bare Cu is *ca.* 55.3° and the contact angle of the cathode coating formed by electrodeposition is *ca.* 154.2° (Fig. 4b) showing super-hydrophobic character. In Fig. 4c, the dynamic behavior of water droplets after hitting the super-hydrophobic surface is mainly divided into three processes: spreading, retracting, and leaving the surface. From Fig. 4c, the water droplets first deform under inertial force and form a pancake shape. Then, as time goes by, the contact diameter gradually increases.

After reaching the maximum contact diameter, the water droplets begin to retract due to the surface tension. Due to the uneven velocity distribution of the water droplets during the retraction process, the water droplet elongates and deforms in the vertical direction, and finally, it leaves the super-hydrophobic surface. In the whole process, the water droplet maintains its integrity. After leaving the super-hydrophobic surface, there is no trace of adhesion of the water droplet on the surface. This phenomenon firstly demonstrates the low interaction between the water droplet and the character of the super-hydrophobic surface. Secondly, if there is some dust on the surface, it will be grabbed by the water droplet to be removed substantially. It is critical to the atmospheric corrosion; when the dirt is on the surface, it will react as the nucleation for generating the water film so that the breakdown will occur with the residence of the liquid film. The self-cleaning property of the super-hydrophobic surface can protect the substrate from contamination, which has significant application value. In displaying the super-hydrophobic surface's self-cleaning behavior, the process of water droplet dropping down as-prepared layering was recorded by high-speed camera pictures. In Fig. 4d, as seen, the droplets of water gently drip on the super-hydrophobic surface with a certain inclination angle, the ZnO particles wrap away with the water droplets in the rolling process. Finally, the water droplets can remove the ZnO particles from the super-hydrophobic surface during the rolling process. Due to the weak affinity between the water solution and the super-hydrophobic matrix, it is expected that the droplets will slip off easily and remove dust on the surface. However, surface tension tends to cause water droplets to adhere to the sphere and encounter the solid, resulting in smaller wet areas and smaller roll angles. As the droplets pass through the super-hydrophobic surface, the water droplets gradually turn pink. The droplets remain spherical during the rolling process, and the dehydrated ZnO powder is collected in the aqueous phase. You can see that the aqueous phase cleans the dirt. Therefore, low surface energy materials have excellent self-cleaning properties.

Meanwhile, its motion activity is not affected. The air remaining in the microstructure gap of the super-hydrophobic surface can reduce the contact area between the water droplets and the matrix.^33^ Therefore, self-cleaning is realized; even the hydrophilic ZnO particle has a minimal size.

**Fig. 5:**
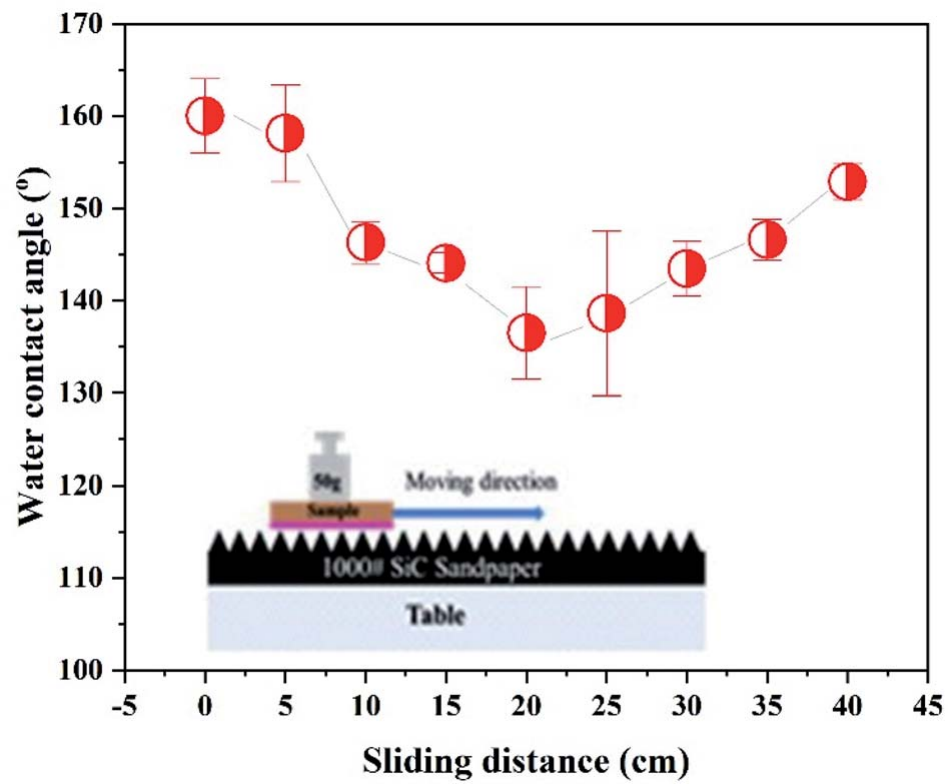
The changing wetting behavior of deposits on Cu corresponds to the increase of abrasion distance.

On the other hand, evaluating the mechanical effect, especially when the super-hydrophobic surface proposed usage, could be in an actual situation. As shown in Fig. 5, an abrasion experiment conducted is to understand the as-fabricated super-hydrophobic surface robustness. Before mechanical abrasion, the super-hydrophobic surface and water droplet contact angle were high as *ca.* 160°. The surface contact angle firstly decreases and then increases with the increase of the rubbing distance. It is because of the multi-scale nanostructures formed on the Cu surface by electrodeposition. After 40 cm of friction, the contact angle maintained was at *ca.* 152.9°, showing excellent superhydrophobicity corresponding to the friction resistance. The above results show better mechanical resistance than other reported super-hydrophobic surfaces recently formed on metal surfaces.^16,34^ The coarse sandpaper partially damaged the microstructure, and the root means a section of the surface roughness was reduced by an order of microns. As a result, the height of the remaining microstructure decreases sharply. However, this can contribute to a reduction in contact angle. Moreover, the surface was still very super-hydrophobic as air entrap into the porous spaces. In summary, even after wear, microscale structures were present on the damaged surface and were reduced in size to maintain good superhydrophobicity.

**Fig. 6:**
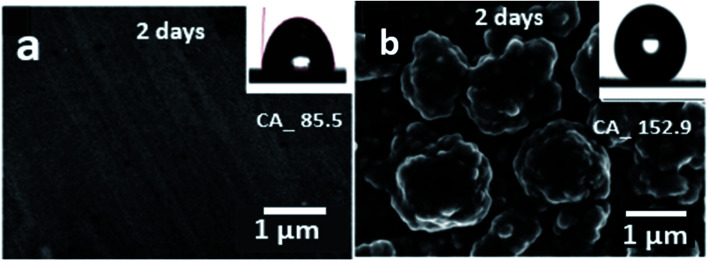
Evaluation of (a) bare Cu and (b) the super-hydrophobic surface immersed in deionized water for 2 days.

A time-dependent experiment was also carried out to evaluate the stability of the coated cobalt myristic surface immersed in deionized water for two days. As-received bare Cu has a smooth surface at a micrometer without apparent surface roughness, reflecting a hydrophilic surface-reaching as high as *ca*. 85.5° (Fig. 6a). The bare Cu tends to maintain the water droplet at a low angle with the increase in spreading than rolling off, while the surface tension brings the water droplet to the sphere to contact the solid, resulting in a low wetting area a low rolling angle. The bare Cu was immersed in water and analyzed under polarizing light to understand the changes in surface morphology, which appeared unaltered. The coated super-hydrophobic surface morphology has no noticeable difference after being immersed in water for 24 hours. As shown in Fig. 6b, the surface wettability of the super-hydrophobic surface was characterized with water static CA to be 152.9°, and the film contained numerous microprotrusions with diameters of ∼1 μm. The space among these microprotrusions is ∼100 nm in size, resulting in many cavities with submicrometer sizes in the film. Thus, the film exhibits a complex morphology on the micro protrusions. Such dual-scale structures are like lotus leaves, which facilitate surface hydrophobicity.^35^ The CA of the super-hydrophobic surface is highly related to the surface roughness, with a stable and robust effect on water, exhibiting the importance of dual-scale roughness for superhydrophobicity.

**Fig. 7:**
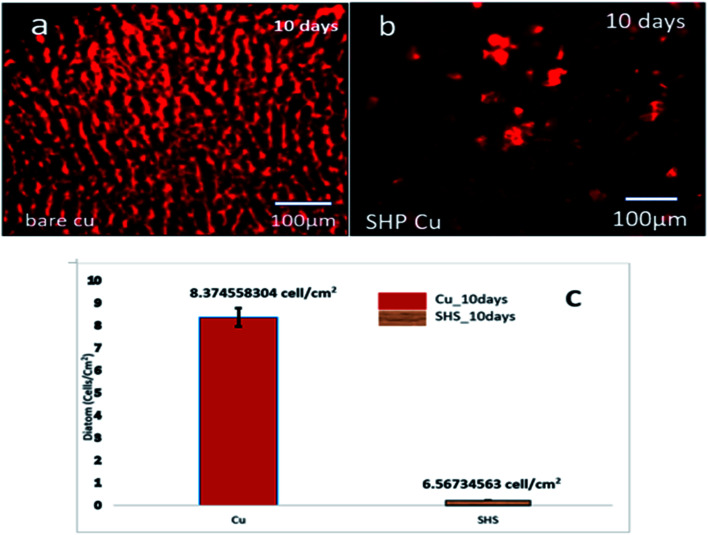
(a) Bare Cu biological adhesion, (b) superhydrophobic Cu biological adhesion, and (c) results of diatom adhesion of the bare Cu and super-hydrophobic Cu after ten days of immersion.

The suspension of diatoms is utilized to investigate the self-cleaning capabilities of the super-hydrophobic coating in more detail, particularly in the presence of biological fluid. The seawater environment is a typical watery habitat that supports a varied range of bio-organisms and microbes. Even though Cu can spontaneously generate the ion required to execute the antifouling capability, the corrosion of Cu is what causes the antifouling ability. It determines whether the super-hydrophobic coating has a bio-adhesion inhibitory impact on diatoms, typical biofouling species in saltwater. When exposed to a fluorescent microscope for ten days, bare Cu substrate becomes covered with red spots, as shown in the image taken by fluorescence microscope (Fig. 7a) (diatoms). Only a few number diatoms can be found to cling to the super-hydrophobic Cu surface. According to the statistical data (as shown in Fig. 7b), the number of diatoms on the bare copper surface can reach as high as *ca.* 8 × 10^10^ cells per cm^2^, whereas the number of diatoms on the super-hydrophobic Cu surface is almost one order of magnitude lower than that on a bare copper surface (*ca.* 6 × 10^8^ cells per cm^2^). The coating still had a solid self-cleaning function after being exposed to saltwater containing diatoms as biofouling organisms. The super-hydrophobic as it has been constructed can be thought of as a composite coating that includes air and solid phases. The bio-organisms will not be able to establish anchor sites in the air. When it comes to anchoring, the organisms can only attempt to anchor on the hydrophobic solid-state protrusion area, which is extremely limited compared to the bare metal substrate. As a result, the amount of biofouling inhibition provided by the super-hydrophobic surface will continuously rise.

**Fig. 8:**
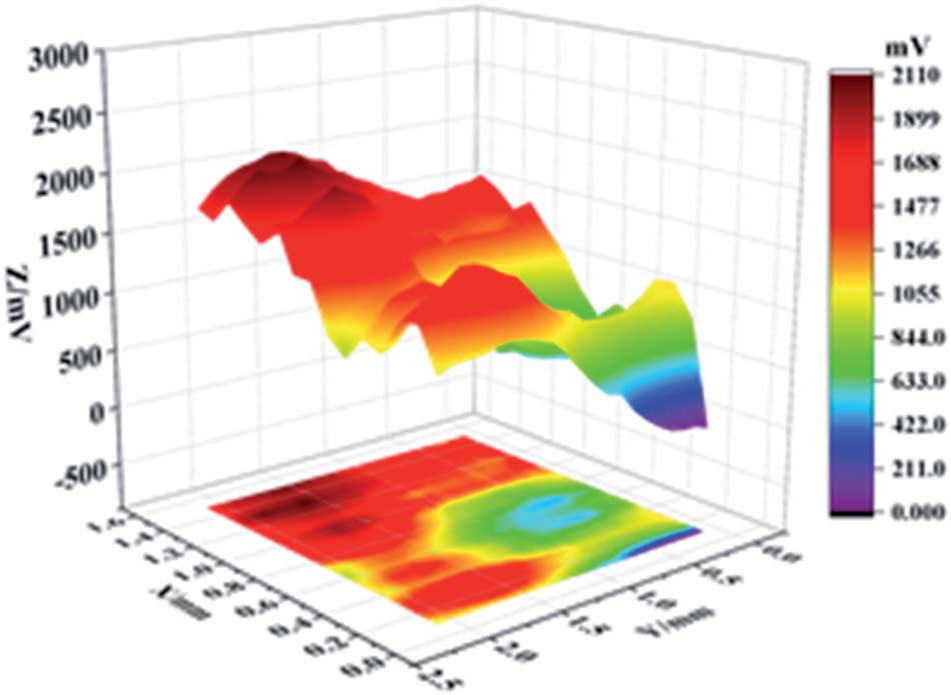
The potential distribution map in the junctional area of bare Cu and Cu-superhydrophobic surface.

In knowing the super-hydrophobic surface's corrosion resistance in the atmospheric condition, the scanning kenning probe (SKP) usage illustrates a designated area covering both bare Cu and Cu coated by super-hydrophobic surface obtain a potential distribution map. Fig. 8 shows the potential distribution diagram of the conjunction area between bare Cu and Cu covered by a super-hydrophobic surface prepared by electrodeposition. The highest and lowest potential of this specific area is −0.29 V and −0.65 V, respectively. Thus, the maximum potential difference between super-hydrophobic surface and bare Cu surface is Δ*E* = 0.36 V.

Therefore, the super-hydrophobic surface enhances the atmospheric corrosion inhibition performance.

**Fig. 9:**
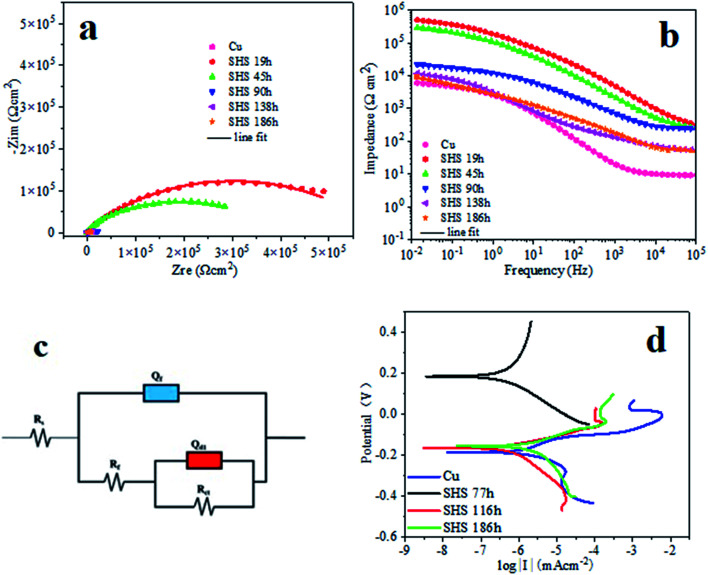
Electrochemical impedance spectroscopy is the method to evaluate the corrosion inhibition of Cu and SHS Cu samples in 3.5 wt% NaCl solution, including (a) Nyquist plots and (b) Bode plots. (c) The fitting electrical circuits correspond to the behavior of experimental EIS data. (d) Potentiodynamic polarization curves of bare Cu and super-hydrophobic Cu samples in 3.5 wt% NaCl solution with different immersion times.

The evaluated corrosion resistance of the electrodeposited super-hydrophobic surface was further using powerful electrochemical impedance spectroscopy (EIS). Fig. 9 shows their corresponding Nyquist plots and Bode plots. As known, the Nyquist loop diameter refers to the working electrode's polarization resistance in the corrosion process. After 19 h of deposition time, the electrodeposited super-hydrophobic surface presented a giant circle due to the protective coating of cobalt and myristic acid, followed by the 45 h electrodeposited cobalt coating. The bare copper substrate had the minor Nyquist loop, indicating that it was subject to corrosion quickly in the NaCl solution than the electrodeposited cobalt and the super-hydrophobic surface. Bode plots also give the corrosion inhibition performance of the coating.

The higher the |*Z*|_0.01Hz_ value, the better the corrosion resistance.^35^Fig. 9b shows the three surfaces' Bode plots (log impedance modulus |*Z*| *versus* log frequency). Noticeably, the bare copper substrate possessed the lowest impedance modulus |*Z*| in the low-frequency range, while the as-fabricated super-hydrophobic surfaces had the largest impedance modulus |*Z*|. After 19 h of soaking, the |*Z*|_0.01Hz_ value of super-hydrophobic surface is *ca.* 5.11 × 10^5^ Ω cm^2^, which is *ca.* two orders of magnitude larger than bare Cu weighting 6.36 × 10^3^ Ω cm^2^. When the immersion time extension was 186 h, the |*Z*|_0.01Hz_ value of SHS dropped to 8.40 × 10^3^ Ω cm^2^, which is still more extensive than bare Cu. The super-hydrophobic surface resistance decreases when immersion time increases because the water phase slowly dissolves the air layer stored in the micro/nanostructure. These results confirm that deposited super-hydrophobic coating can perform excellent corrosion resistance to protect the bare copper material.

To further analyze the impedance data of these three samples, a twin equivalent circuit was applied to fit the Nyquist plots (as shown in Fig. 9c). It shows that the equivalent circuit diagrams of bare Cu and super-hydrophobic surfaces are the same. But in this circuit, the resistance (*R*_f_) for bare Cu refers to the corrosion product film. Meanwhile, the super-hydrophobic surface relates to the super-hydrophobic film layer (including the air layer). There is no corrosion product film because the corrosion of Cu covered by the super-hydrophobic surface is negligible. *R*_s_ and *R*_ct_ represent solution resistance and charge transfer resistance, respectively. *Q*_d1_ and *Q*_f_ refer to constant phase angle components, which simulate the capacitance of the electric double layer and the film, respectively. The formula defines the impedance of the continuous phase angle element:

4 *Z*_CPE_ = *Y*_0_^−1^ (*jω*)^−*n*^

Among them, *Y*_0_ is the constant phase angle element coefficient related to the capacitance. Parameter *j* is the unit of the imaginary part, *ω* is the angular frequency (rad s^−1^), and *n* between 0 and 1 is a dimensionless factor. In addition, this experiment uses charge transfer resistance to evaluate the corrosion inhibition efficiency, namely:

5 *η*_1_ (%) = (1 − *R*^0^_ct_/*R*_ct_) × 100

In formula (5), *R*^0^_ct_ represents the charge transfer resistance of the bare Cu, and *R*_ct_ represents the charge transfer resistance of the super-hydrophobic surface.

Table 1 shows the electrochemical parameters obtained by fitting electrochemical impedance spectroscopy. As observed in Table 1 that *Q*_dl_ and *R*_ct_ of bare Cu are 3.10 × 10^−5^ F cm^−2^ and 2.81 × 10^4^ Ω cm^2^, respectively. For super-hydrophobic surface, *Q*_dl_ and *R*_ct_ become (1.57–3.56) × 10^−6^ F cm^−2^ and (1.27–5.80) × 10^5^ Ω cm^2^, respectively. Thus, the *Q*_dl_ of bare Cu is higher than that of the super-hydrophobic surface, and the *R*_ct_ is smaller than that of the super-hydrophobic surface, which is about one order of magnitude, which means that bare Cu is more prone to let charge transfer than the super-hydrophobic surface. According to eqn (5), the calculation gives that the corrosion inhibition efficiency in the initial stage of the prepared super-hydrophobic surface is as high as 95.16%.

**Table 1:** Electrochemical parameters revealed by EIS of Cu and super-hydrophobic surface samples immersed in 3.5 wt% NaCl solution

Samples	*R*_s_ (Ω cm^2^)	*Q*_f_ (F cm^2^)	*n*_f_	*R*_f_ (Ω cm^2^)	*Q*_d1_ (F cm^2^)	*n*_d1_	*R*_ct_ (Ω cm^2^)	*η*_1_ (%)
Cu	9.23 × 10^0^	1.85 × 10^−4^	0.80	1	3.10 × 10^−5^	0.87	2.81 × 10^4^	—
SHS 19 h	2.22 × 10^2^	2.75 × 10^−7^	0.80	3.73 × 10^4^	1.65 × 10^−6^	0.80	5.80 × 10^5^	95.16
SHS 45 h	2.08 × 10^2^	9.42 × 10^−7^	0.80	5.02 × 10^4^	3.56 × 10^−6^	0.80	3.63 × 10^5^	92.26
SHS 90 h	1.68 × 10^2^	2.15 × 10^−5^	0.38	7.92 × 10^1^	1.57 × 10^−6^	0.76	2.47 × 10^5^	88.62
SHS 138 h	4.71 × 10^1^	5.77 × 10^−5^	0.80	2.99 × 10^2^	3.31 × 10^−6^	0.80	1.77 × 10^5^	84.12
SHS 186 h	2.57 × 10^1^	1.59 × 10^−4^	0.37	4.01 × 10^0^	2.03 × 10^−6^	0.82	1.68 × 10^5^	83.27

Therefore, the super-hydrophobic surface prepared by one-step electrodeposition can effectively protect the Cu metal and slow down corrosion. The reason is that the super-hydrophobic surface obtained in the experiment can form an effective barrier on the Cu substrate due to its excellent super-hydrophobic property, hindering the diffusion of corrosive ions to the material. Thus, it has a super anti-corrosion effect on the metal substrate of Cu.

Moreover, Fig. 9d shows the polarization curves of the super-hydrophobic surface after being immersed in 3.5 wt% NaCl solution for 77 h, 116 h, and 186 h, respectively. The coating's corrosion potential (*E*_corr_) and corrosion current density (*I*_corr_) originated from the potentiodynamic polarization curve, as listed in Table 2. The *E*_corr_ and *I*_corr_ of the bare copper are −0.19 V and 9.21 × 106 A cm^−2^, respectively. However, after being immersed for 77 h in the solution, the corrosion current densities of the super-hydrophobic surface were found to be 1.09 × 10^−7^ A cm^−2^, which is one order of magnitude smaller than the bare copper, which is 9.21 × 10^−7^ A cm^−2^. When the immersing time further increases, from 77 h to 116 h, the *I*_corr_ of the coating increases to 7.39 × 10^−7^ A cm^−2^. However, it is still lower than that of bare copper. Furthermore, when the immersion time increases to 186 h, the corrosion current density of super-hydrophobic surface increases significantly to 2.66 × 10^−6^ A cm^−2^ than the 77 h and 116 h but 1/3 smaller than the bare Cu, still showing high corrosion resistance, which demonstrated that the corrosion resistance of bare copper substrate was significantly enhanced owing to the presence of the electrodeposited super-hydrophobic cobalt myristic coating. In general, the higher corrosion potential and lower corrosion current density indicated that the layer has superior corrosion resistance.

**Table 2:** Electrochemical parameters fitted from the potentiodynamic polarization curves of bare Cu and SHS in a 3.5 wt% NaCl solution

Samples	*E*_0_ (V)	*I*_0_ (A cm^−2^)	*β*_a_ (mV per decade)	−*β*_c_ (mV per decade)	*η*_2_ (%)
Cu	−0.19	9.21 × 10^−6^	68.28	39.51	—
SHS 77 h	0.18	1.09 × 10^−7^	870.69	169.17	98.82
SHS 116 h	−0.16	7.39 × 10^−7^	62.26	154.95	91.98
SHS 186 h	−0.16	2.66 × 10^−6^	77.10	199.31	71.12

In addition, we also use the corrosion current density to evaluate the corrosion inhibition efficiency, namely:

6 *η*_2_ (%) = (1 − *I*_SHS_/*I*_Cu_) × 100

The corrosion inhibition efficiency calculated from the corrosion current density is 98.82%, 91.98%, and 71.12% after being immersed in 3.5 wt% NaCl solution for 77 h, 116 h, and 186 h, respectively, confirming that the super-hydrophobic surface prepared in this experiment has excellent corrosion resistance.

## 4 Conclusion

This experiment successfully constructed a copper-based super-hydrophobic surface by a one-step electrodeposition method with improved corrosion resistance. The surface component is mainly cobalt myristate. The SEM images indicate that the copper surface became much rough with tight micro/nanoscale spherical clusters after the deposition of cobalt film. Such unique surface texture can contribute to trapping a large amount of air, and the air cushion would prevent the water droplet from penetrating the copper surface, showing a super-hydrophobic property. In one-step electrodeposition, the deposition of the cobalt myristate complex makes the surface have multiple degrees of layer structure with low surface energy, so the character has excellent super-hydrophobic properties, and its static properties contact angle is *ca.* 152.9°. The results of surface chemistry confirm that the comprising super-hydrophobic surfaces were of cobalt crystals (Co) and cobalt myristate crystals (Co[CH_3_(CH_2_)_12_COO]_2_).

Furthermore, the self-cleaning test confirmed that the prepared super-hydrophobic surface has very low adhesion, and the water droplets cannot stay on the surface and are easy to roll. Through electrochemical experiments in 3.5 wt% NaCl solution, the super-hydrophobic surface has the following conclusions:

(1) The corrosion inhibition efficiency of the super-hydrophobic surface prepared in this experiment is high as 98.92%.

(2) After 19 h of soaking in 3.5 wt% NaCl solution, the |*Z*|_0.01Hz_ value of super-hydrophobic surface is about 5.11 × 10^5^ Ω cm^2^, two orders of magnitude greater than bare copper (6.36 × 10^3^ Ω cm^2^).

Therefore, the assumption is that the corrosion performance was consistent with surface wettability because the super-hydrophobic surface can prevent the corrosive ions (Cl^−^) from contacting the bare metal surface. Furthermore, the electrodeposition process is straightforward and fast. Thus, it is effectively producing a super-hydrophobic surface with excellent corrosion inhibitive properties on various metals.

## Conflicts of interest

The authors proclaim that they have not identified competing of financial interests or personal relationships that could have impacted the work reported in this article.

## Supplementary Material

RA-012-D1RA06473H-s001
