# Supplementary material for: Bioinspired super-hydrophobic fractal array via a facile electrochemical route: preparation and corrosion inhibition for Cu
Source: RSC Adv. 2021 Dec 20;12(1):265–76. doi: 10.1039/d1ra06473h (PMC8978658; doi:10.1039/d1ra06473h)
Supplement: RA-012-D1RA06473H-s001 [file RA-012-D1RA06473H-s001.pdf]

Electronic Supplementary Material (ESI) for RSC Advances.
This journal is © The Royal Society of Chemistry 2021

Supplementary file

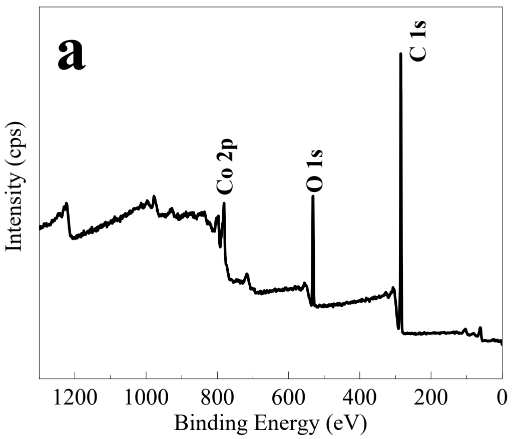

Fig 2a. (a) XPS survey spectra of Cu covered with the deposit obtained by electrolysis at 10 V for 20 min
